# The Tomb of Secrets

**Published:** 1864-07

**Authors:** 


THE TOMB OF SECRETS.

We read that "a physician in Paris was accused, on the 11th inst., before a French Police Court, of having revealed the 'secret disease' of one of his patients, and that to the patient's damage. He was found guilty, and condemned by the court to pay a fine of 500 francs, and to undergo a year's imprisonment. Moreover, at the expiration of his imprisonment, he was to remain for five years under the surveilance of the police, and to pay all the expenses of the trial. Besides this, having injured the patient's reputation, he was ordered to pay the patient 1000 francs as damages."

There can be no doubt that this vigorous administration of the law was conceived in the very best spirit, even though part of the punishment is one not known to English jurists, except in the instance of men possessing diplomas of a different class—tickets of leave. Imprisonment and fine, in cases of libel, have, ere this, been imposed by English judges; but "surveilance of the police" is an institution with which we are practically unacquainted, but which all who are aware of the French system know to imply complete social ruin. It is our intention, however, to treat rather with the principles than with the particulars. The French law is jealous of professional confidence. It recognises the social necessity of preserving in the highest perfection the good faith and inviolable integrity of the medical practitioner. English legislation, generally speaking, prefers the practical appeal to the pocket, and seeks not so much to punish as to compensate—making the damages awarded the measure of the penalty, unless in cases where the judge, according to the nature of "the information," deems the offence rather of a criminal than of a civil character.—*Lancet.*

